# Time to retire the serial Papez circuit: Implications for space, memory, and attention

**DOI:** 10.1016/j.neubiorev.2022.104813

**Published:** 2022-09

**Authors:** John P. Aggleton, Andrew J.D. Nelson, Shane M. O’Mara

**Affiliations:** a School of Psychology, Cardiff University, 70 Park Place, Cardiff CF10 3AT, Wales, UK; b School of Psychology and Trinity College Institute of Neuroscience, Trinity College Dublin, The University of Dublin, Dublin D02 PN40, Ireland

**Keywords:** Amnesia, Attention, Context, Hippocampus, Mammillary bodies, Memory, Space, Thalamus

## Abstract

After more than 80 years, Papez serial circuit remains a hugely influential concept, initially for emotion, but in more recent decades, for memory. Here, we show how this circuit is anatomically and mechanistically naïve as well as outdated. We argue that a new conceptualisation is necessitated by recent anatomical and functional findings that emphasize the more equal, working partnerships between the anterior thalamic nuclei and the hippocampal formation, along with their neocortical interactions in supporting, episodic memory. Furthermore, despite the importance of the anterior thalamic for mnemonic processing, there is growing evidence that these nuclei support multiple aspects of cognition, only some of which are directly associated with hippocampal function. By viewing the anterior thalamic nuclei as a multifunctional hub, a clearer picture emerges of extra-hippocampal regions supporting memory. The reformulation presented here underlines the need to retire Papez serially processing circuit.

## Highlights

• Why it is time for the concept of Papez circuit to be retired.
• The anterior thalamic nuclei and hippocampus conjointly support episodic memory.
• Focus should now shift to how these two structures jointly interact with neocortex.
• Some anterior thalamic functions are independent of the hippocampus.
• The anterior thalamic nuclei provide a multifunctional hub.

**Fig. 1:**
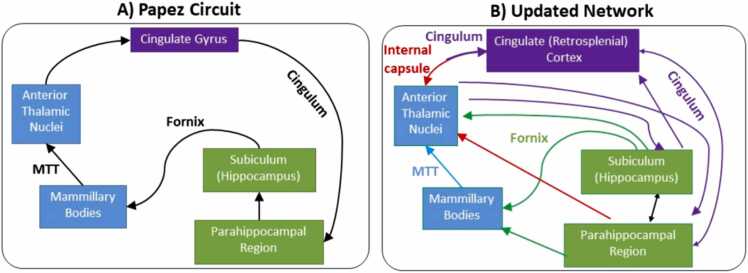
A) Schematic of the principal connections described by Papez (1937) that constitute a serial circuit for the experience of emotions. B) Updated schematic depicting major, direct interconnections between the sites in Papez circuit, based on axonal tracing data from the rat and nonhuman primate. The schematic also provides information on the pathways linking these structures. Blue arrows, fibres in mammillothalamic tract; green arrows, fibres in fornix; purple arrows, fibres in cingulum bundle; red arrows, fibres in internal capsule. (Note that the parahippocampal projections to the rat anterior thalamic nuclei join the internal capsule, while the corresponding projections in the macaque involve the fornix). Abbreviations: MTT, mammillothalamic tract.

For many, the first encounter with the anterior thalamic nuclei comes from their inclusion within Papez circuit for emotion (Fig. 1A). This classic theory proposed *‘that the hypothalamus, the anterior thalamic nuclei, the gyrus cinguli, the hippocampus and their interconnections constitute a harmonious mechanism which may elaborate the functions of central emotion’* (Papez, 1937). This first encounter is a mixed blessing. As a systems model of affective processing, Papez’ circuit is fundamentally flawed (Dalgleish, 2004). This same model did, however, bring into prominence various limbic structures that are serially linked and seemingly functionally integrated, namely the hippocampal formation, mammillary bodies, anterior thalamic nuclei, cingulate cortices, and parahippocampal cortices. The concept of Papez circuit remains highly influential (e.g., Choi et al., 2019; Ji et al., 2020; Forno et al., 2021). One prominent example is how this circuit is increasingly viewed as critical for long-term memory. Here, we show how this idea emerged and why both that memory model and Papez circuit now need radical restructuring.

The initial section of this review describes some of the clinical findings that promoted the idea that Papez circuit supports episodic memory - our memory for unique day-to-day events. The extent of evidence helps to explain why such models persisted for over 50 years (Delay and Brion, 1969, Aggleton and Brown, 1999, Aggleton and Brown, 2006, Vertes et al., 2001, Wang et al., 2018, Ji et al., 2020). We also describe how the classic Papez circuit conception has been superseded by axonal tracing data showing that at its best it is a simplification and, at worst, highly misleading. We then argue that while the hippocampus and anterior thalamic nuclei support each other, their relationship is not hierarchical. Consequently, the hippocampus and anterior thalamic nuclei have different functions, some of which are expressed by their direct interconnections, others by their individual connections, and others by their convergent actions on neocortical sites. From these insights, we replace the classic serial circuit with a tripartite model of episodic memory that emphasises how the hippocampal formation and anterior thalamic nuclei separately and together act upon neocortical sites to ensure the encoding and retrieval of episodic memories.

As part of this reformulation, we show that the anterior thalamic nuclei support a variety of functions (including mnemonic, spatial, and aspects of attention), some of which appear to be independent of the hippocampus. These multiple functions encompass the different properties of the principal anterior thalamic nuclei, i.e., the anterodorsal nucleus (AD), the anteromedial nucleus (AM), and the anteroventral nucleus (AV). In making the case for this new perspective, we consider the following:

i) Whether structures and pathways comprising Papez circuit are vital for memory.
ii) Whether Papez circuit is serial.
iii) Hippocampal functions that require the anterior thalamic nuclei.
iv) Anterior thalamic nuclei functions that require the hippocampal formation.
v) The multiple cognitive contributions of the anterior thalamic nuclei: From context to attention.
vi) How different properties of the principal anterior thalamic nuclei contribute to different aspects of cognition.
vii) How anterior thalamic - neocortical interactions promote memory and attention.

We conclude that adhering to the classic Papez circuit occludes rather than facilitates anatomical and functional understanding of these complex circuits, and it is now time to retire that model in favour of a more modern conception rooted in the latest anatomical and functional data. [Note that throughout, the terms hippocampal formation and hippocampal refer to the dentate gyrus, CA fields, and subiculum (Burwell and Witter, 2002). The presubiculum, parasubiculum, and postsubiculum are placed within the parahippocampal region. The term ‘amnesia’ refers to anterograde amnesia.].

i) *Whether structures and pathways comprising Papez circuit are vital for memory.*
  The importance of the hippocampus for episodic memory has been confirmed by its necessary involvement in temporal lobe amnesia (e.g., Scoville and Milner, 1957; Rempel-Clower et al., 1996; Spiers et al., 2001; Clark and Squire, 2010; Lad et al., 2019). The corresponding situation for diencephalic amnesia has, however, proved far more complex. Despite frequent reports of mammillary body atrophy in this condition (Delay and Brion, 1969, Dusoir et al., 1990, Tsivilis et al., 2008, Carlesimo et al., 2011, Kopelman, 2015), a range of other diencephalic sites are variably involved (e.g., Mair et al., 1979; Markowitsch, 1982; Zoppelt et al., 2003; Cipolotti et al., 2008). Despite this variability, the key cognitive features of temporal lobe amnesia and diencephalic amnesia (impaired long-term memory, spared IQ, spared nondeclarative learning, and spared short-term memory) are highly similar (Aggleton, 2008), as are rates of forgetting (e.g., McKee and Squire, 1992; Kopelman and Stanhope, 1997). Unsurprisingly, the alcoholic Korsakoff syndrome can present with additional features reflecting its more widespread pathology (Kopelman, 2015).
  Starting from the hippocampus, we follow the classic serial Papez circuit to review the importance of each component pathway and structure for memory. Immediately beyond the hippocampus is the fornix (Fig. 1A). This pathway contains many different hippocampal connections including those reaching the mammillary bodies and anterior thalamic nuclei (Poletti and Creswell, 1977, Aggleton, 2012). Fornix damage causes amnesia (e.g., Gaffan and Gaffan, 1991; D'Esposito et al., 1995; McMackin et al., 1995; Aggleton et al., 2000; Vann et al., 2008; Takano et al., 2018; Salvalaggio et al., 2018), often impairing the recall of information more than the recognition of previously experienced information (McMackin et al., 1995, Aggleton et al., 2000, Vann et al., 2008, Vann et al., 2009b; see also Adlam et al., 2009; Jonin et al., 2018). Added evidence from diffusion MRI studies shows that indices of fornix integrity correlate with episodic memory (Rudebeck et al., 2009, Metzler-Baddeley et al., 2011, Hodgetts et al., 2017), including an association with recollective but not familiarity-based recognition (Rudebeck et al., 2009). Other diffusion MRI analyses highlight the significance of the link between the subiculum and the fornix for episodic memory (Hartopp et al., 2019), which is notable as the subiculum is the primary source of hippocampal projections to sites including the mammillary bodies and anterior thalamic nuclei (Bubb et al., 2017). Limitations with the above findings include how the fornix links the hippocampal formation with a wide range of sites beyond the diencephalon (Poletti and Creswell, 1977), while also containing both hippocampal efferent and afferent fibres (Lewis and Shute, 1967).
  A far more selective disconnection follows damage to the mammillothalamic tract, the next pathway in Papez circuit. This tract emanates from the mammillary bodies to enter the rostral thalamus. The projections within this tract are unidirectional and only appear to terminate in the anterior thalamic nuclei. Several analyses have contrasted those thalamic stroke patients with persistent memory loss against those with preserved memory. These analyses all conclude that pathology in a rostral thalamic zone centred around the mammillothalamic tract provides the best predictor of memory loss (Von Cramon et al., 1985, Van der Werf et al., 2000, Carlesimo et al., 2011, Kopelman, 2015).
  Related insights come from studies of third ventricle colloid cyst patients. In one such study, mammillary body volume repeatedly correlated with episodic memory recall, although this relationship was not found for recognition memory (Tsivilis et al., 2008). Further analyses revealed a sparing of familiarity-based recognition, contrasting with a loss of recollection-based recognition (Vann et al., 2009b). Thus, like the fornix findings (e.g., Aggleton et al., 2000; Rudebeck et al., 2009) the mammillothalamic connections appear vital for the recall of episodic information but not for some forms of recognition (Carlesimo et al., 2007).
  Additional evidence comes from the many post-mortem studies of the amnesic Korsakoff’s syndrome. While mammillary body atrophy is always observed (Delay and Brion, 1969, Victor et al., 1971), the presence of other pathologies has made it difficult to isolate the contributions of individual diencephalic sites (Kopelman et al., 2015). However, detailed cellular comparisons between alcoholic Korsakoff amnesics and alcoholic Wernicke encephalopathy cases (with no persistent amnesia) revealed that pathology in the anterior thalamic nuclei may be the best predictor of the severe memory loss in this syndrome (Harding et al., 2000). This conclusion was reinforced by imaging analyses of Korsakoff’s syndrome, which indicated that atrophy of the hippocampal input zone in the anterior thalamus is a characteristic feature of the memory loss (Segobin et al., 2019). Such findings do not, however, preclude the contributions of other thalamic nuclei to this complex cognitive disorder (Mair et al., 1979, Kopelman, 2015, Segobin et al., 2019).
  The next Papez pathway is the cingulum bundle (Fig. 1A). This complex tract contains fibres from numerous brain sites, including the anterior thalamic projections to the cingulate and retrosplenial cortices, as well as to the hippocampal formation (Domesick, 1970, Shibata, 1993a, Shibata, 1993b; Heilbronner and Haber, 2014; Bubb et al., 2018, 2020). Despite the array of connections contributing to the cingulum, there is little neuropsychological evidence linking cingulum bundle damage with persistent memory loss (Bubb et al., 2018). Many of the null findings come from patients with interruption of the bundle at the level of the anterior cingulate cortex (Bubb et al., 2018). These results are, however, difficult to interpret as anterior thalamic efferents and afferents take a range of routes that join, leave, and cross the cingulum bundle at different rostral-caudal levels (Domesick, 1970, Bubb et al., 2018, Bubb et al., 2020). For this reason, an effective disconnection of Papez circuit would require extensive pathology along the length of this tract. Such pathology would most almost inevitably invade retrosplenial cortex, which is itself strongly associated with anterograde amnesia (Valenstein et al., 1987, Maguire, 2001, Vann et al., 2009a, Ferguson et al., 2019). Thus, it has proved very difficult to isolate the impact of caudal cingulum bundle damage. At the same time, the importance of retrosplenial cortex for episodic memory further links Papez circuit to memory loss.
  In summary, there is considerable evidence that pathways and sites along Papez circuit, including the anterior thalamic nuclei, are vital for human long-term memory. Some of these studies highlight the significance of these same connections for the recall, rather than the recognition, of episodic memory. Together, this body of neuropsychological evidence explains how the concept of a Papez memory circuit emerged and persisted.
ii) *Papez circuit is not a serial circuit.*
  The image of a serial circuit based on that described by Papez (Fig. 1A) remains hugely influential (Aggleton and Brown, 2006, Choi et al., 2019, Forno et al., 2021). One reason is that the described connections undoubtedly exist. Even so, this same image has promoted some persistent misconceptions. The traditional depiction of this ‘circuit’ reinforces the view that sites such as the anterior thalamic nuclei are downstream, i.e., subsidiary to the hippocampal formation. This view is reinforced by the monosynaptic connections in the mammillary bodies, closely linking the hippocampal formation with the anterior thalamic nuclei (Umaba et al., 2021). Terms such as ‘extended hippocampal system’, which include the anterior thalamic nuclei (Aggleton and Brown, 1999), again unwittingly imply that these diencephalic sites are of secondary importance.
  A second misconception is the notion of a unidirectional return circuit. Of the connections described by Papez, only two are unidirectional – the projections from the hippocampal formation to the mammillary bodies (via the fornix) and the projections from the mammillary bodies to the anterior thalamic nuclei (via the mammillothalamic tract). While Fig. 1A depicts the original circuit described by Papez (1937), the adjacent figure provides an updated depiction based on known connections in the rat and nonhuman primate (Fig. 1B, see Bubb et al., 2017). This updated version, which incorporates various reciprocal connections, shows how the anterior thalamic nuclei and cingulate cortices occupy more central positions, rather than appearing as satellites of the hippocampal formation. In addition, the anterior thalamic nuclei receive *direct* inputs from the hippocampal formation and parahippocampal region, which rely on the fornix in nonhuman primates (Aggleton et al., 1986, Saunders et al., 2005). These anatomical updates (Fig. 1B) underline the complexity of the supposed ‘circuit’ and belie the idea of unidirectional processing in favour of a more networked set of interactions.
  One striking feature of the revised schematic is how retrosplenial cortex (areas 29, 30) receives convergent, dense inputs from both the hippocampal formation and the anterior thalamic nuclei in both rodents and primates (Kobayashi and Amaral, 2003, Shibata and Yukie, 2003, Bubb et al., 2017). All three anterior thalamic nuclei have dense, reciprocal connections with the retrosplenial cortex that are topographically organised (Sripanidkulchai and Wyss, 1986, van Groen et al., 1993, Shibata, 1993b, Bubb et al., 2017). For example, the rat dysgranular cortex (area 30) is more interconnected with AM, while the granular cortex (area 29) is especially interlinked with AD and AV (van Groen et al., 1993, Bubb et al., 2017, Aggleton et al., 2021, Lomi et al., 2021). Meanwhile, the anterior cingulate cortex is principally interconnected with AM (Shibata, 1993b, Shibata and Naito, 2005).
  The retrosplenial cortex also receives dense hippocampal inputs (Kobayashi and Amaral, 2003, Bubb et al., 2017), thereby opposing the traditional direction of Papez circuit. In nonhuman primates there is a spread of termination across areas 29 and 30 (Kobayashi and Amaral, 2003, Aggleton et al., 2012), while in rodents the corresponding hippocampal projections heavily target area 29 (granular retrosplenial cortex). Excitatory inputs arise from the dorsal subiculum, alongside much lighter inhibitory projections from CA1, which both target area 29 (Yamawaki et al., 2019a, Yamawaki et al., 2019b). In rodents, many of the same subiculum neurons that innervate area 29 have collaterals that also innervate the mammillary bodies (Kinnavane et al., 2018), fundamentally undermining the concept of a serial circuit. Meanwhile, few retrosplenial neurons project directly to the hippocampal formation, instead relying on indirect routes via parahippocampal areas and the anterior thalamic nuclei (Sugar et al., 2011, Prasad and Chudasama, 2013, Bubb et al., 2017).
  A further limitation with Papez circuit is that it classically portrays a closed loop. This image downplays the significance of structures that interact with Papez circuit but are located outside the loop. Relevant sites include the prefrontal and anterior cingulate cortices, which are closely connected both anatomically (Shibata and Kato, 1993, Shibata and Naito, 2005, Wright et al., 2013, Xiao and Barbas, 2002, Prasad and Chudasama, 2013) and functionally (Gabriel et al., 1983, Gabriel, 1993, Bubb et al., 2021) with sites including the anterior thalamic nuclei. Subcortical influences include ascending tegmental inputs that are vital for mammillary body and anterior thalamic function (Mitchell et al., 2002, Bassett et al., 2007, Vann, 2013, Vann and Nelson, 2015). Perhaps the best-known example concerns the head-direction information that the lateral mammillary nucleus conveys to the anterior thalamic nuclei and, thence, to parahippocampal and hippocampal sites (Taube, 2007). Information helping to drive this navigational compass comes from the dorsal tegmental nucleus of Gudden (Bassett et al., 2007, Dillingham and Vann, 2019), i.e., extrinsic to Papez circuit.
  These same considerations lead us to next describe how some hippocampal functions depend on the integrity of the anterior thalamic nuclei via these extrinsic connections. In contrast with Papez serial circuit, some of these functions reflect direct thalamo-hippocampal projections.
iii) *Hippocampal functions that require the anterior thalamic nuclei.*
  Animal studies reveal multiple ways in which the anterior thalamic nuclei potentially support hippocampal function, most of which relate to spatial memory. This focus reflects how anterior thalamic lesions are sufficient to cause severe, persistent deficits across a wide range of hippocampal-dependent spatial tasks (Aggleton and Nelson, 2015, Clark and Harvey, 2016). These tasks include spatial alternation (Aggleton et al., 1995, Aggleton et al., 1996, Warburton et al., 1999, Savage et al., 2011), radial-arm maze foraging (Aggleton et al., 1996, Byatt and Dalrymple-Alford, 1996, Moran and Dalrymple-Alford, 2003, Mitchell and Dalrymple-Alford, 2005, Mitchell and Dalrymple-Alford, 2006), automated delayed nonmatching-to-position (Aggleton et al., 1991), path integration (Frohardt et al., 2006), location learning in a water-maze based on distal room cues (e.g., Sutherland and Rodriguez, 1989; Warburton and Aggleton, 1998; Moreau et al., 2013; Wolff et al., 2008), and location learning based on geometric cues (Aggleton et al., 2009, Dumont et al., 2014b).
  While these findings point to close parallels between the anterior thalamic nuclei and the hippocampus, they do not confirm an obligatory relationship. That evidence first came from cross-hemispheric disconnection lesion studies (Warburton et al., 2001, Henry et al., 2004), but this technique does not isolate the direction of effect nor does it distinguish between direct and indirect interactions. Consequently, chemogenetic methods were used to target the direct anterior thalamic projections to the dorsal hippocampal formation (Nelson et al., 2020). Inhibiting these direct projections disrupted spatial working memory when rats could not use local maze cues (Nelson et al., 2020).
  Explanations for why the hippocampal formation might require anterior thalamic inputs include: a) providing spatial information for the hippocampal formation and parahippocampal region, b) optimising hippocampal plasticity, and c) enabling nonspatial information extrinsic to Papez circuit to reach the hippocampus. These same explanations do not, however, reflect the totality of anterior thalamic functions.
  a) Head-direction cells, which provide a compass-like signal, are numerous within AD (Taube, 1995, Taube, 2007), although subsequent studies have also found them in AV (Tsanov et al., 2011a). This directional information, which reaches the anterior thalamic nuclei from the lateral mammillary body nucleus (Blair et al., 1999, Hopkins, 2005), is necessary for head-direction firing in the hippocampal formation and parahippocampal region (Goodridge and Taube, 1997, Winter et al., 2015, Frost et al., 2021). This same thalamic information also appears necessary for normal grid-cell activity in parahippocampal areas (Winter et al., 2015). It might, therefore, be assumed that the loss of the anterior thalamic head-direction signal would be sufficient to produce a cascade of persistent, severe spatial deficits.
    In fact, the loss of head-direction information is not a sufficient explanation for why the anterior thalamic nuclei are so vital for spatial memory. This conclusion is backed by a variety of evidence (Aggleton et al., 2010, Dillingham and Vann, 2019, Dudchenko et al., 2019). For example, lateral mammillary body lesions (which block head-direction information reaching the anterior thalamus) only have mild, sometimes just transient, effects on spatial learning (Vann, 2005, Vann, 2018), in marked contrast to the persistent, severe deficits following anterior thalamic lesions. Indeed, complete lesions of the mammillary bodies (lateral and medial nuclei combined), as well as lesions of the mammillothalamic tract, still have a lesser impact than anterior thalamic lesions (Aggleton et al., 1991, Aggleton et al., 1995, Perry et al., 2018). Meanwhile, selective lesions targeting the individual anterior thalamic nuclei repeatedly show that all three nuclei (AD, AM, AV) contribute to spatial learning (Aggleton et al., 1996; Byatt et al., 1996; van Groen et al., 2002; Safari et al., 2020), even though AD is the principal source of hippocampal/parahippocampal head-direction information and might, therefore, be expected to be the most critical.
    In addition to head-direction, some anterior thalamic neurons show spatial responses that closely resemble the place, grid, and border cells described in medial temporal areas (O‘Mara and Aggleton, 2019). One important question is what happens to hippocampal spatial signalling (beyond head-direction) after the loss of anterior thalamic inputs. In one such study, CA1 place cells retained their location-specific firing (although there was a loss of spatial coherence and information content), leading to greater place-field instability between sessions (Calton et al., 2003). Another study observed an apparent preservation of CA1 place cells after complete anterior thalamic lesions (Frost et al., 2021), although lateral mammillary lesions were sufficient to disrupt the discrimination by place cells of similar locations from different orientations (Harland et al., 2017).
    Perhaps a more striking effect following anterior thalamic lesions is the apparent loss of all types of spatially-responsive signalling (place, head direction, grid, and border cells) within the subiculum (Frost et al., 2021), in addition to chance-level performance on a spatial memory task. It is difficult to ascribe these widespread effects to a selective loss of head-direction signals. Rather, they might reflect how anterior thalamic inputs (either directly or indirectly) modulate gating actions within the subiculum (Frost et al., 2021). Gating has, for example, been seen as a means to segregate individual blocks of to-be-remembered data (Raghavachari et al., 2001) or to disengage task-irrelevant pathways (Jensen and Mazaheri, 2010).
  b) A second explanation for the importance of the anterior thalamic nuclei concerns their potential significance for hippocampal plasticity. Relevant findings include a reduction in spine density in hippocampal CA1 neurons following both anterior thalamic (Harland et al., 2014) and mammillothalamic tract (Dillingham et al., 2019) lesions. Other relevant effects following anterior thalamic lesions include hippocampal hypoactivity, as measured by the immediate-early gene c-*fos* and phosphorylated CREB (Jenkins et al., 2002a, Jenkins et al., 2002b, Dumont et al., 2012).
    Electrophysiological analyses have often focussed on theta, reflecting its importance for hippocampal plasticity, enabling processes such as separating epochs, synchronising plasticity, and promoting hippocampal-cortical coherence (Vertes, 2005, Tsanov and Manahan-Vaughan, 2009, Korotkova et al., 2018). A subpopulation of anterior thalamic neurons, especially those within AV, display theta modulation (Vertes et al., 2001, Albo et al., 2003, Tsanov et al., 2011a, Tsanov et al., 2011b), with the hippocampus being one source of this rhythmic activity (Vertes et al., 2001). Following anterior thalamic lesions, rats show only modest changes in the CA1 theta band, alongside clearer evidence for a prefrontal cortex experience-dependent power change in the theta band, as well as a reduction in power change at lower theta frequencies in the dentate gyrus (Ulrich et al., 2019). Meanwhile, inactivation of the medial mammillary nucleus (which projects to AM and AV) reduces the amplitude of hippocampal theta in anaesthetized rats (Żakowski et al., 2017). Mammillothalamic tract damage also lowers hippocampal theta frequency, but can cause excessive inter-regional coherence, affecting both theta and gamma (Dillingham et al., 2019).
    Complementary insights arise from applying oscillatory stimulation to the thalamus. Remarkably, optogenetic theta-burst stimulation of the rat anterior thalamic nuclei can ameliorate the spatial working memory deficit seen after mammillothalamic tract lesions (Barnett et al., 2021). This finding highlights the potential significance of anterior thalamic theta and may open new avenues for the amelioration of memory disorders by invasive (deep-brain stimulation) and non-invasive means (e.g., focal ultrasound; Nunez-Elizalde et al., 2022). In the context of the present review, an important question concerns the extent to which nonhippocampal sources influence anterior thalamic theta. One potential source arises from their ascending tegmental inputs (Shibata, 1992, Vann, 2013, Dillingham et al., 2015b, Dillingham et al., 2019).
    Additional information comes from studies of epileptic patients (Sweeney-Reed et al., 2021). There is, for example, evidence that deep-brain stimulation of the anterior thalamic nuclei can alleviate some intractable epilepsies (Salanova et al., 2018; Yu et al., 2018). One account is that high-frequency stimulation of the anterior thalamus can desynchronize background electrical activity in the ipsilateral hippocampal formation over a broad frequency range and may, thereby, reduce epileptic discharges, including interictal spikes and high-frequency oscillations (Yu et al., 2018).
    Taking advantage of these clinical procedures, working memory precision in epileptic patients was compared for trials with or without anterior thalamic stimulation (Liu et al., 2021). Anterior thalamic stimulation improved colour recall performance, as well as increasing the spectral power of gamma (30–100 Hz) oscillations and decreasing interictal epileptiform discharges (IED) in the hippocampus. Furthermore, increased gamma power during the pre-stimulus and retrieval period predicted this task improvement (Liu et al., 2021). These findings point to effects on cortical and hippocampal plasticity while also showing that these thalamic nuclei can contribute to a range of cognitive domains, not just long-term memory.
  c) The anterior thalamic nuclei provide a route to the hippocampal formation from sites that may not *directly* reach the hippocampus. Such afferents also have the potential to drive differences between anterior thalamic and hippocampal functions. Candidate cortical areas include the prelimbic and anterior cingulate cortices. These frontal areas have disynaptic links via the anterior thalamic nuclei to the dorsal hippocampus (Prasad and Chudasama, 2013) yet few, if any, direct inputs to the hippocampal formation (Jones and Witter, 2007; but see Bian et al., 2019). As indirect prefrontal – hippocampal interactions are increasingly presumed to guide memory retrieval (Eichenbaum, 2017), the anterior thalamic nuclei (along with nucleus reuniens) may have a significant role (Mathiasen et al., 2020). Other anterior thalamic relays that reach the dorsal hippocampus arise in retrosplenial cortex (Prasad and Chudasama, 2013), which also has very few direct projections to the hippocampal formation (Sugar et al., 2011). As already noted, ascending subcortical inputs, e.g., from various tegmental nuclei may also affect hippocampal functions via the anterior thalamic nuclei (Mitchell et al., 2002) or, more indirectly, via the mammillary bodies (Vann, 2013, Dillingham et al., 2015b). Finally, the reciprocal interactions between the thalamic reticular nucleus and the anterior thalamic nuclei (Gonzalo-Ruiz et al., 1995; Lozsádi, 1995) may constrain cortico-thalamic activity (Takata, 2020).
iv) *Anterior thalamic nuclei functions that require the hippocampal formation.*

The classic image of Papez circuit depicts anterior thalamic function as dependent on the hippocampal projections reaching the mammillary bodies via the fornix. To this, we can add the many direct projections from the hippocampal formation (especially the subiculum) to the anterior thalamic nuclei (Fig. 1B). As already described, studies implicating these hippocampal inputs include comparisons with fornix lesion effects. Such experiments show that the severity of anterior thalamic spatial deficits in rats is frequently comparable to that seen after fornix lesions (Aggleton et al., 1991, Aggleton et al., 1995, Warburton and Aggleton, 1998, Warburton et al., 1999). Indeed, in some studies, the anterior thalamic lesion deficit is more severe (Warburton and Aggleton, 1998, Aggleton et al., 2009), potentially reflecting the added significance of anterior thalamic actions on the hippocampal formation.

More direct evidence comes from a recent study that targeted the direct projections from the rat dorsal hippocampal formation (subiculum) to the anterior thalamic nuclei (AV and AM) (Nelson et al., 2020), i.e., not via the mammillary bodies. Chemogenetic inhibition of these direct projections impaired spatial working memory (Nelson et al., 2020). These findings complement the results of surgically lesioning the part of the descending postcommissural fornix that conveys hippocampal inputs to the mammillary bodies (Vann et al., 2011). The resulting mild spatial deficits not only point to the significance of the direct projections from the hippocampal formation to the anterior thalamic nuclei, but also highlight how the mammillary bodies provide critical ascending information that is independent of the hippocampus (Dillingham et al., 2015b, Vann and Nelson, 2015). Meanwhile, diffusion imaging in humans has also helped to separate the precommissural fornix (which reaches frontal and striatal areas) from the postcommissural fornix (which reaches the mammillary bodies and anterior thalamus). Postcommissural fornix indices are more strongly associated with visual recall (Christiansen et al., 2016a) and the learning of spatial configurations (Coad et al., 2020).

Again, there may be multiple reasons for the importance of the hippocampal projections to the anterior thalamic nuclei. These reasons include: a) providing both spatial and nonspatial information for the anterior thalamic nuclei, b) moderating thalamic plasticity, and c) creating opportunities for hippocampal efferents to influence additional distal sites. While some of the effects are undoubtedly via the mammillary bodies (as in Papez circuit) others are direct, while others may be via sites such as retrosplenial cortex.

**Fig. 2:**
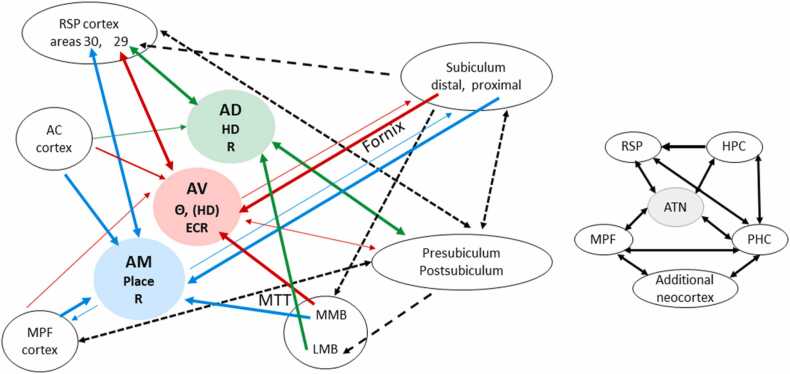
Blue arrows show principal connections of the anteromedial (AM), red arrows the anteroventral (AV), and green arrows the anterodorsal (AD) thalamic nucleus. The black dashed arrows refer to potentially important connections not directly involving the anterior thalamic nuclei (ATN). The thickness of the arrows reflects differences in the density of the projections. The panel to the right provides a thumbnail of key connections. Abbreviations: HD, presence of head-direction units (in parenthesis if lower frequency); θ, theta modulated cells; Place, place cells; impairments in the Morris water maze at encoding (E), consolidation (C), retrieval (R) (from Safari et al., 2020). Other abbreviations: AC, anterior cingulate; HPC, hippocampal formation; LMB, lateral mammillary nucleus; MPF, medial prefrontal cortex; MMB, medial mammillary nucleus; MPF, medial prefrontal cortex; PHC, parahippocampal region; RSP, retrosplenial cortex.
  The key assumption – that the individual nuclei have differing functions - gains support from both selective lesion studies in rodents and the differences in the distributions of spatially-responsive neurons within this region (Jankowski et al., 2015). Lesion studies have largely focussed on spatial tasks. One repeated finding is that selective damage to an individual nucleus causes only mild spatial learning deficits in rats, deficits that become appreciably more severe as more anterior thalamic nuclei are involved (Aggleton et al., 1996; Byatt et al., 1996; van Groen et al., 2002; Safari et al., 2020). The conclusion is that no individual nucleus is responsible for the severe spatial deficits seen after anterior thalamic lesions.
  Of the three nuclei, AM provides the principal hub linking prefrontal cortex with hippocampal sites. These connections point to potential roles in memory retrieval such as combatting interference, as well as regulating aspects of attention based on reward history (Bubb et al., 2021). The close association of the rodent AM with the proximal subiculum (closest to CA1) and with perirhinal cortex further suggests a bias to object-based information. At the same time, recent studies point to the location of engram cell ensembles relating to contextual fear conditioning within this nucleus (Roy et al., 2022a, Roy et al., 2022b). Meanwhile, AV provides a strategic node between retrosplenial cortex and the hippocampus (Gabriel, 1993; Yamawaki et al., 2019b). Its affiliation with the distal subiculum (Christiansen et al., 2016b) further underlines its likely role in enabling the effective use of spatial information (Kitanishi et al., 2021), including associative contextual information. This same nucleus is the principal site of theta modulated neurons in the anterior thalamic nucleus, implicating AV in both retrosplenial and hippocampal plasticity (Vertes et al., 2001, Yamawaki et al., 2019b). Its importance for contextual-fear conditioning (Yamawaki et al., 2019b) and for the maintenance of spatial memory (Safari et al., 2020, Roy et al., 2022b) further highlight its broader significance. Meanwhile, AD is principally concerned with navigation and the ways in which orientation information feeds into a variety of spatial metrics. It may also moderate long-term retrieval processes (Vetere et al., 2021). Contributing to these various functions are the mammillary bodies. While AD receives its inputs from the lateral mammillary nucleus, different neurons within the medial mammillary nucleus project to AM and AV (Hopkins, 2005, Wright et al., 2013).
  Further evidence comes from the transient inactivation of individual thalamic nuclei. Inactivation of AV impaired the acquisition, consolidation, and retrieval phases of the Morris water-maze task (Safari et al., 2020) while also disrupting the retention phase of a spatial working memory task (Roy et al., 2022b), results indicative of a pervasive spatial mapping deficit. This interpretation matches how AV is highly interconnected with other spatial loci within the brain, including the dorsal hippocampal formation (Shibata, 1993a) (Fig. 2). In contrast, transient AD and AM inactivation only affected the retrieval phase of the spatial location task (Safari et al., 2020; see also Vetere et al., 2021). Underlying the wider spatial contributions of AV, a context passive-avoidance task was disrupted during consolidation (AM, AV inactivation) and retrieval (only AV inactivation) (Safari et al., 2020).
c) *How anterior thalamic - neocortical interactions promote memory and attention.*
  By highlighting the co-dependency of the anterior thalamic nuclei and hippocampal formation there is the potential danger of re-inventing Papez serial circuit. That can be dismissed on numerous grounds. Perhaps most telling is that many functions reflect reciprocal connections within Papez circuit or act in the opposite direction, e.g., hippocampal formation on retrosplenial cortex. The presence of so many subiculum neurons that collaterise to innervate both the mammillary bodies and retrosplenial cortex further undermines the classic model (Kinnavane et al., 2018). At the same time, there is considerable evidence that the anterior thalamic nuclei and hippocampal formation, in addition to influencing each other, separately act with cortical areas within and beyond Papez circuit to enable cognition.
  As observed, contextual fear conditioning has been used to investigate parallel anterior thalamic/hippocampal actions on retrosplenial cortex. One set of experiments in mice established that the inhibitory projections from hippocampal area CA1 to layer I of granular retrosplenial cortex (area 29) oppose the actions of the AV inputs to the same cortical area, which promote contextual fear conditioning (Yamawaki et al., 2019b). Meanwhile, separate classes of excitatory efferents from the dorsal subiculum to granular retrosplenial cortex may differentially support recent and long-lasting fear conditioning (Yamawaki et al., 2019a). These findings highlight the importance of the convergent actions of the anterior thalamic nuclei and hippocampal formation on a third site, e.g., retrosplenial cortex (Wolff and Vann, 2019, Yamawaki et al., 2019b, Aggleton and O’Mara, 2022). At the same time, computation modellers have highlighted the benefit of convergent actions from hippocampal and thalamic engram cells on the consolidation of cortical engrams (Tome et al., 2022), set alongside evidence that cells in the anterior thalamic nuclei contribute to network engram cells (Roy et al., 2022a). Thereby a picture emerges of the significance of the conjoint, but distinct, actions of the anterior thalamic nuclei and hippocampal formation on those cortical sites that they jointly influence (Fig. 3) – actions vital for normal memory consolidation and retrieval.

a) To help understand the potential significance of the hippocampal (subiculum) projections to the anterior thalamic nuclei for spatial and nonspatial signalling, it is useful to consider the topography of these efferents. In the rat the ‘proximal’ subiculum (nearest to CA1) preferentially projects to AM, while the distal subiculum preferentially projects to AV (Meibach and Siegel, 1977, Christiansen et al., 2016b). This separation is informative because the lateral entorhinal and perirhinal cortices favour the proximal subiculum while the medial entorhinal and postrhinal cortices favour the distal subiculum (Witter et al., 2000). This organisation suggests that the proximal subiculum (which targets AM) preferentially processes object-based information while the distal subiculum (which targets AV) processes spatial-based information (Kim et al., 2012; Cembrowski et al., 2018). This same bias might, therefore, be anticipated in the functions of AM (objects) and AV (space).
  Up to now, electrophysiological studies have largely focussed on spatial signalling. In addition to head-direction, the anterior thalamic nuclei contain neurons responsive to other classes of spatial information (O‘Mara and Aggleton, 2019). For example, the dorsal subiculum appears to provide place information for AV, signalling associated with theta oscillations (Kitanishi et al., 2021). Meanwhile, both place cells and border/perimeter cells have been found in AM (Jankowski et al., 2015, Matulewicz et al., 2019).
  The anterior thalamic nuclei also receive projections from the postsubiculum and presubiculum, sites that contain many head-direction cells. These inputs predominantly terminate within AD, as well as the laterodorsal thalamic nucleus (van Groen and Wyss, 1990a, van Groen and Wyss, 1990b). In rodents, many of these projections join the internal capsule rather than the fornix (Dillingham et al., 2015a). While postsubiculum lesions do not eliminate AD head-direction signalling, consistent with the principal importance of ascending pathways (Taube, 2007), they can moderate AD activity so that visual landmarks exert less control over preferred firing direction (Goodridge and Taube, 1997). While the loss of these parahippocampal influences could have a modest impact on spatial learning, it could not explain, for example, how silencing dorsal hippocampal (subiculum) projections to AM and AV impairs spatial alternation (Nelson et al., 2020).
b) The second explanation concerns the potential contributions of hippocampal efferents to thalamic plasticity. As already noted, one focus has been on the role of theta, with studies emphasising how hippocampal theta might resonate through Papez circuit (Vertes et al., 2001, Vertes, 2005). Consistent with this view, most theta-related cells in the anterior thalamic nuclei fire at higher rates in the presence of hippocampal theta (especially in AV), although a small proportion (in AM and AD) reduce their firing rates (Albo et al., 2003). In addition, the amplitude of anterior thalamic theta spectral power was found to increase in rats after stimulation of the fornix (Tsanov et al., 2011d). While low-frequency stimulation of the fornix depressed anterior thalamic synaptic responses (Tsanov et al., 2011c, Tsanov et al., 2011d), low-frequency stimulation of the mammillothalamic route resulting in anterior thalamic activity that correlated positively with the fast band of thalamic theta oscillation (Tsanov et al., 2011d). Furthermore, anterior thalamic long-term depression (LTD) was induced after stimulation of its direct fornical connections while long-term potentiation (LTP) was induced predominantly through the mammillothalamic tract route (Tsanov et al., 2011c, Tsanov et al., 2011d). These findings help to contrast direct hippocampal influences on the anterior thalamic nuclei with those that might arise more independently from the mammillary bodies (Vann, 2013, Dillingham et al., 2015b, Dillingham and Vann, 2019). These same results also provide insights into why the anterior thalamic nuclei should not be considered as ‘relays’.
c) A third explanation reflects how the anterior thalamic nuclei might provide indirect routes for hippocampal influences beyond Papez circuit. Candidate targets include the anterior cingulate cortex, which receives appreciable anterior thalamic inputs, especially from AM (Shibata, 1993b, van Groen et al., 1999). In contrast, the light hippocampal inputs to the rat anterior cingulate cortex are concentrated close to the border with prelimbic cortex (Jay and Witter, 1991) while in the monkey they seem largely restricted to just area 24a (Aggleton et al., 2015). Consequently, links via the anterior thalamic nuclei may provide wider frontal influences. Part of this function may be to assist in the propagation of theta modulation and spatial signalling from the subiculum (Vertes et al., 2001, Kitanishi et al., 2021). These same anterior thalamic efferents also highlight the scope for actions independent of the hippocampal formation.

a) *The multiple cognitive contributions of the anterior thalamic nuclei: From context to attention.*
  While the anterior thalamic nuclei clearly contribute to navigation, they have a much broader role in supporting cognition. One key example concerns ‘context’ information as models of episodic memory repeatedly emphasise the contributions of context both for the encoding and retrieval of long-term memory (Diana et al., 2007, Ranganath and Ritchey, 2012, Barry and Maguire, 2019). ‘Context’ is often a proxy for place (and/or time), representing the constellation of external, background stimuli within which an event occurs. Meanwhile, the term ‘cue’ often refers to a single distinctive element, e.g., a light or auditory stimulus.
  Like hippocampal lesions, anterior thalamic nuclei damage can disrupt contextual fear conditioning but spare cued fear conditioning (Dupire et al., 2013, Marchand et al., 2014, de Lima et al., 2017, Lopez et al., 2018). Strikingly, anterior thalamic and hippocampal CA1 projections on retrosplenial cortex both influence contextual fear conditioning but have opposing actions (Yamawaki et al., 2019b). While anterior thalamic inputs facilitate learning, those from CA1 are inhibitory (Yamawaki et al., 2019b). These competing actions are highly informative, in part because the hippocampal effects run counter to the direction of Papez circuit, but also because the anterior thalamic actions appear independent of the hippocampus.
  Other learning tasks further highlight how the anterior thalamic nuclei are important for using arrays of distal cues to distinguish spatial contexts. For example, anterior thalamic nuclei lesions impair biconditional discriminations (in context A, stimulus X is reinforced, Y is not reinforced; in context B, stimulus X is not reinforced, Y is reinforced) when the contextual stimuli comprise *distal spatial* cues (Dumont et al., 2014a; see also Sziklas and Petrides, 1999) but spare contextual conditional discriminations involving *local, elemental* stimuli such as different visual, thermal or floor texture cues (Dumont et al., 2014a; see also Wolff et al., 2015). Other studies indicate that the anterior thalamic nuclei help contextual information to overcome interference (Law and Smith, 2012) and disambiguate competing task information (Kinnavane et al., 2019).
  A different form of context concerns temporal information. Like the hippocampus and medial prefrontal cortex, lesions of the rodent anterior thalamic nuclei impair the ability to discriminate between objects or odours based on relative recency (Wolff et al., 2006, Albasser et al., 2012, Dumont and Aggleton, 2013, Barker et al., 2017). However, the anterior thalamic involvement in recency judgements appears more restricted than that of the hippocampus, with deficits only emerging when animals make fine-grained temporal discriminations between multiple items (Wolff et al., 2006, Albasser et al., 2012, Barker and Warburton, 2011, Dumont and Aggleton, 2013). Evidence that temporal cues might preferential engage distal CA1 (Beer et al., 2018), points to a potential link with the proximal subiculum and the anteromedial nucleus. Together, these findings reveal multiple ways in which contextual information (spatial and temporal) can be used by the anterior thalamic nuclei to serve episodic memory.
  The anterior thalamic nuclei make further contributions to cognition that extend beyond contextual processing (Nelson, 2021). One source of evidence comes from landmark animal studies that revealed anterior thalamic and cingulate cortex interactions during both aversive and appetitive discrimination learning (Gabriel et al., 1977, Gabriel et al., 1983, Gabriel, 1993, Gabriel and Talk, 2001, Smith et al., 2002). Neurons in AV and the cingulate cortex selectively increase their firing rate in response to presentations of a stimulus predictive of footshock (S+), a pattern of discriminatory activity that typically emerged in later stages of learning (Gabriel et al., 1977). Furthermore, AV lesions abolish discriminative neuronal firing in the anterior cingulate and retrosplenial cortices (Gabriel et al., 1983). Also, combined anterior thalamic and mediodorsal thalamic lesions in rabbits led to greater acquisition deficits of a signalled avoidance task than lesions restricted to the mediodorsal thalamic nucleus (Gabriel et al., 1989). The authors concluded that the anterior thalamic nuclei, in partnership with cingulate cortices, support associative attention to reward-related cues, a role most evident during later stages of discrimination learning (Gabriel, 1993, Gabriel and Talk, 2001).
  Further evidence has since emerged of a more specific role in attention. Rats with anterior thalamic lesions (Wright et al., 2015) were first trained on a series of nonspatial discriminations in which the same category of cues consistently predicted reward while another cue category was unrelated to reward (Birrell and Brown, 2000). Normal rats increasingly attend to the rewarded stimulus dimension, so that learning accelerates over successive discriminations, reflecting the acquisition of a cognitive ‘intradimensional-set’. Next, for the first time, the previously irrelevant stimulus category predicts reward (‘extradimensional-shift’). This shift retards learning while rats re-align their attention (Birrell and Brown, 2000). In contrast to controls, anterior thalamic nuclei lesions *impaired* learning across the initial successive discriminations, failing to show evidence of an ‘intradimensional-set’ (Wright et al., 2015). The same lesions then *facilitated* learning the switch to the stimulus dimension that had hitherto been irrelevant (the ‘extradimensional-shift’) (Wright et al., 2015). The implication is that the anterior thalamic nuclei normally mediate attention to task relevant stimuli that are reliable predictors of reward, enabling intradimensional-sets at the expense of extradimensional-shifts. The lesions enhanced category switching as these animals remained attending to stimuli that had previously been poor predictors of reward.
  This learning profile is the mirror-image of that seen after medial prefrontal lesions (Dias et al., 1996; Birrell et al., 2000). Furthermore, hippocampal lesions appear to produce a profile of deficits similar to that seen after prefrontal lesions, i.e., qualitatively different to that following anterior thalamic lesions (Marquis et al., 2008). Importantly, evidence that the human anterior thalamic nuclei have similar roles in attention emerges from clinical and imaging data (Bourbon-Teles et al., 2014, Leszczyński and Staudigl, 2016), albeit at lower anatomical resolution to that from animal studies. Meanwhile, other animal lesion studies help to show that the anterior thalamic nuclei are not needed for all aspects of attention (Chudasama and Muir, 2001, Mantanona et al., 2020).
  Findings from human fMRI research support the view that the anterior thalamic nuclei have diverse cognitive actions. Isolating these nuclei with fMRI does, however, remain challenging, and so some fMRI studies incorporate adjacent thalamic nuclei (e.g., Bourbon-Teles et al., 2014; Pergola et al., 2013; Kim and Maguire, 2019). Nevertheless, studies of navigation show increased anterior thalamic BOLD signals associated with head rotation (Kim and Maguire, 2019). Moving to learning and memory, there are descriptions of increased anterior thalamic BOLD signals during the retrieval of learnt information (Pergola et al., 2013, Antonucci et al., 2021), while fMRI studies of recognition memory appear consistent with the prediction that the anterior thalamic nuclei assist recall-based recognition rather than familiarity-based recognition (Kafkas et al., 2020, Geier et al., 2020). In one of these studies, the reduction of anterior thalamic activity during the encoding phase of an associative recognition memory task predicted subsequent successful performance (Geier et al., 2020). In the other, recollection, but not familiarity, was correlated with anterior thalamic activity (Kafkas et al., 2020).
  A meta-analysis of thalamic fMRI studies across a broad range of cognitive tasks found that within the thalamus, the anterior thalamic nuclei, the mediodorsal nucleus, and the intralaminar nuclei, contributed the most to overall cognitive processing across multiple domains (Antonucci et al., 2021). This pattern appears consistent with the concept of a ‘multi-demand’ network (Duncan, 2010). This fMRI evidence for a broad category of anterior thalamic functions also matches the conclusion from a study of focal thalamic lesions in human patients (Hwang et al., 2021). Again, both the anterior thalamic nuclei and the mediodorsal thalamic nucleus emerged as hubs supporting multiple aspects of cognition, including executive functions, memory, and language (Hwang et al., 2021). This multiplicity of effects may reflect their widespread thalamo-cortical connections.
b) *How different properties of the principal anterior thalamic nuclei contribute to different aspects of cognition.*
  The three major anterior thalamic nuclei have different properties (Fig. 2). The connectivity of AD is the most distinct as it has a narrow array of targets, with a heavy bias to parahippocampal areas (Fig. 2). Meanwhile, despite many overlapping sources of afferents and shared targets, systematic differences can be observed between AM and AV (Wright et al., 2013, Christiansen et al., 2016b). These differences are further maintained by how individual neurons infrequently collaterise to innervate both nuclei (Wright et al., 2013). Gene expression studies have also put the three nuclei in distinct groupings (Phillips et al., 2019). While AM and AV were placed in separate, multinuclear groupings, AD had a unique thalamic profile (Phillips et al., 2019).

**Fig. 3:**
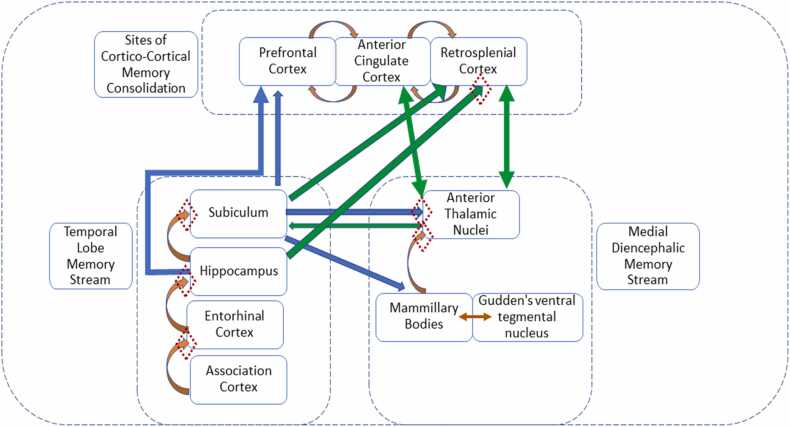
Activity-dependent synaptic plasticity in parallel, interacting circuits supports memory consolidation across three distinct but interacting locales: a temporal lobe memory stream; a medial diencephalic memory stream; and sites of cortico-cortical memory consolidation. Note, retrosplenial cortex also projects to the hippocampus, via the parahippocampal region. Diamonds indicate previously-observed sites of synaptic plasticity; other synapses remain to be comprehensively investigated.
  A very different behavioural approach was used to study anterior thalamic – anterior cingulate interactions. Building on how anterior thalamic lesions in rats impair intradimensional-set formation but facilitate extradimensional-shifts (Wright et al., 2015), attention turned to the anterior cingulate cortex. Inhibiting the anterior cingulate cortex produced a pattern of intradimensional-set (impaired) and extradimensional-shift (facilitated) performance (Bubb et al., 2021), similar to that after anterior thalamic lesions. Moreover, selectively disrupting anterior cingulate terminals in AM and AV, impaired the rats’ ability to solve successive discriminations involving a constant stimulus dimension (intradimensional-set), while at the same time facilitating extradimensional-shifts (Bubb et al., 2021), i.e., mimicked anterior thalamic lesion effects. As previously noted, hippocampal lesions produce a very different profile of changes on this task (Marquis et al., 2008). These findings help to show how the anterior thalamic nuclei have vital attentional functions in collaboration with the anterior cingulate cortex, functions that do not reflect hippocampal actions.
  Helping to bring these ideas together, recording studies in epileptic patients have described activity in the anterior thalamic nuclei, as well as the mediodorsal thalamic nucleus, that can predict memory performance (Sweeney-Reed et al., 2014, Sweeney-Reed et al., 2016). For example, aspects of theta oscillations and their synchrony across neocortical-anterior thalamic areas, including cross-coupling with gamma oscillations, predicted subsequent memory for complex photographic scenes (Sweeney-Reed et al., 2014). In a further study, phase amplitude coupling between theta (4–6 Hz) phase and high frequency band (80–150 Hz) amplitude thought to facilitate the coordination of neural oscillatory activity required for cognitive processing, was consistently seen in the anterior thalamic nuclei during rest, but then modulated by task performance (Sweeney-Reed et al., 2017). Meanwhile, when performing a recognition test, theta (4–8 Hz) power in the anterior thalamic nuclei was found to increase for old compared to new items (Bauch et al., 2018). One interpretation is that the anterior thalamic nuclei aid the selection of local, task-relevant high frequency activity associated with specific memory trace attributes (Sweeney-Reed et al., 2021). These thalamic actions are presumed to involve information from widespread neocortical sources, including frontal areas (Sweeney-Reed et al., 2021).
d) *Summary*

**Fig. 4:**
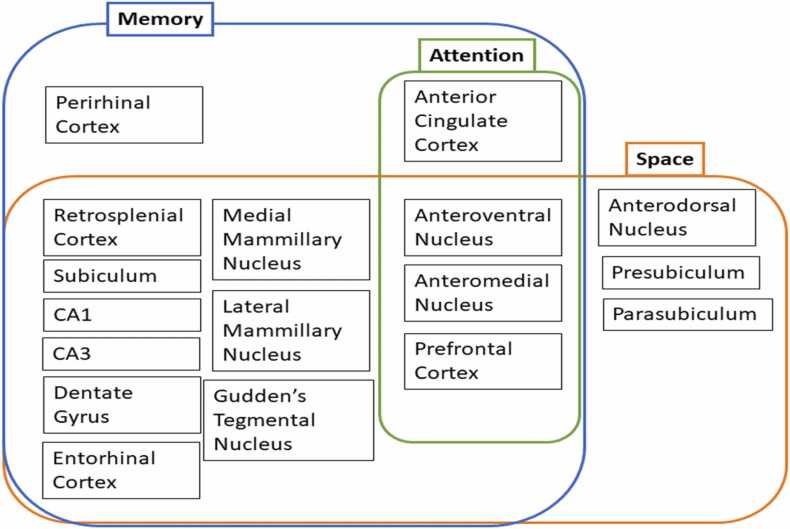
Proposed anterior thalamic participation in cognition. Hypothesised mapping of three interleaving, interconnected, ‘cognitive zones’ of anterior thalamic nuclear influence. Each zone maps cognitive functions — spatial processing, attention, and memory to anterior nuclei (as implicated by lesion, recording, anatomical tracing, or other evidence) illustrating their extensive contributions to multiple aspects of cognition. This mapping of functions implies fast-acting, dynamic relations between spatial processing, attentional, and mnemonic processes; spatial processing and attention are deployed during episodic memory encoding and consolidation (as they are contributory processes to memory), but equally they may well be independent of memory during other tasks.
6) To understand the multiple contributions of the anterior thalamic nuclei to cognition, greater focus should be given to their prefrontal and cingulate interconnections, along with their ascending tegmental inputs.

A series of conclusions emerge from this analysis:

1) While the term ‘Papez circuit’ summarises a set of known connections, it remains misleading for many reasons. We argue that these connections comprise far more than a serial circuit and critically involve interactions beyond the classic circuit. Hence, this phrase should pass into neuroanatomical history. Instead, we need to broaden our conceptions because the complex connectivity of Papez circuit contradicts notions of unidirectional processing in favour of interconnected and networked anatomical structures. The term ‘circuit’ has unwittingly created misleading assumptions about the relationships between the anterior thalamic nuclei and hippocampal formation, consequently inhibiting progress in understanding how differing brain systems may work together and separately to facilitate important aspects of cognition.
2) The anterior thalamic nuclei partner the hippocampal formation, i.e., they are not simply driven by hippocampal actions. Nor are the anterior thalamic nuclei merely relays. Rather, the two sites have different functions, yet they support each other. This balance helps to explain why the anterior thalamic nuclei and hippocampus are, respectively, key loci in the pathology of diencephalic and temporal lobe amnesia.
3) Attention should be given to the convergent actions of the hippocampal formation and anterior thalamic nuclei on neocortical sites (Fig. 3). One such site is the retrosplenial cortex where both anterior thalamic and hippocampal interactions may regulate the production, subsequent re-creation, and flexible use of context within long-term memory.
4) Sites and pathways within Papez original circuit are more vital for the recall, rather than the recognition, of episodic information. This difference reflects how familiarity information is detected in structures beyond these components of the classic circuit.
5) The anterior thalamic nuclei support diverse aspects of cognition (Fig. 4), consistent with acting as a multi-functional hub, e.g., for memory, space, and attention. Only some of these functions are in partnership with the hippocampal formation. These multiple functions partly reflect the different contributions of the individual anterior thalamic nuclei.

Analyses of the anterior thalamic nuclei in humans increasingly show how they may contribute to a wide array of cognitive functions. Both neuropsychological and fMRI findings indicate roles in attention and memory (Bourbon-Teles et al., 2014, Geier et al., 2020). Meanwhile, direct stimulation of the anterior thalamic nuclei can influence working memory performance (Liu et al., 2021). Meta-analyses of fMRI data further highlight how these nuclei appear to contribute to multiple cognitive domains (Antonucci et al., 2021). This conclusion is supported by a comparison of cases with focal thalamic lesions (Hwang et al., 2021) that revealed sites, including the anterior thalamic nuclei, that affect executive functions, memory, and language. One implication is that prefrontal cortical sites interact with the anterior thalamic nuclei to enable not only long-term memory (Sweeney-Reed et al., 2021) but also other cognitive functions, some of which are likely to be independent of the hippocampus. At the same time, the value of studying the anterior thalamic nuclei is underlined by growing evidence that these nuclei contribute to age-related changes in cognition (Roy et al., 2022b) including the early stages of dementias, such as Alzheimer’s disease (Braak and Braak, 1991, Aggleton et al., 2016, Forno et al., 2021). Consequently, there should be renewed efforts to appreciate these thalamic nuclei and their varied cognitive functions in both healthy and diseased brains (Aggleton and O’Mara, 2022; Wolff and Vann, 2019).

## Data Availability

where the data refer to the authors own work it will always be shared on request.
